# Cannabidiol Impairs Brain Mitochondrial Metabolism and Neuronal Integrity

**DOI:** 10.1089/can.2022.0011

**Published:** 2023-03-30

**Authors:** Christopher D. Drummond-Main, Younghee Ahn, Mitchell Kesler, Cezar Gavrilovici, Do Young Kim, Ivana Kiroski, Samantha L. Baglot, Amy Chen, Keith A. Sharkey, Matthew N. Hill, G. Campbell Teskey, Jong M. Rho

**Affiliations:** ^1^ Cell Biology & Anatomy, University of Calgary, Calgary, Canada.; ^2^ Hotchkiss Brain Institute, University of Calgary, Calgary, Canada.; ^3^ Department of Pediatrics, University of Calgary, Calgary, Canada.; ^4^ Alberta Children Hospital Research Institute, University of Calgary, Calgary, Canada.; ^5^ Department of Neurosciences, University of California San Diego, Rady Children's Hospital, San Diego, San Diego, California, USA.; ^6^ Department of Pediatrics, University of California San Diego, Rady Children's Hospital, San Diego, San Diego, California, USA.; ^7^ Department of Pharmacology, University of California San Diego, Rady Children's Hospital, San Diego, San Diego, California, USA.; ^8^ Barrow Neurological Institute, St. Joseph's Hospital and Medical Center, Phoenix, Arizona, USA.; ^9^ Department of Physiology & Pharmacology, University of Calgary, Calgary, Canada.; ^10^ Snyder Institute for Chronic Diseases, University of Calgary, Calgary, Canada.

**Keywords:** cannabidiol, epilepsy, toxicology, neurobiology, pharmacology

## Abstract

**Background:**

The mechanisms underlying the clinical effects of CBD remain poorly understood. Given the increasing evidence for CBD's effects on mitochondria, we sought to examine in more detail whether CBD impacts mitochondrial function and neuronal integrity.

**Methods:**

We utilized BE(2)-M17 neuroblastoma cells or acutely isolated brain mitochondria from rodents using a Seahorse extracellular flux analyzer and a fluorescent spectrofluorophotometer assay. Mitochondrial ion channel activity and hippocampal long-term potentiation were measured using standard cellular electrophysiological methods. Spatial learning/memory function was evaluated using the Morris water maze task. Plasma concentrations of CBD were assessed with liquid chromatography–mass spectrometry, and cellular viability was evaluated using the 3-(4,5-dimethylthiazol-2-yl)-2,5-diphenyltetrazolium bromide (MTT) reduction neuronal injury assay.

**Results:**

At low micromolar concentrations, CBD reduced mitochondrial respiration, the threshold for mitochondrial permeability transition, and calcium uptake, blocked a novel mitochondrial chloride channel, and reduced the viability of hippocampal cells. These effects were paralleled by *in vitro* and *in vivo* learning/memory deficits. We further found that these effects were independent of cannabinoid receptor 1 and mitochondrial G-protein-coupled receptor 55.

**Conclusion:**

Our results provide evidence for concentration- and dose-dependent toxicological effects of CBD, findings that may bear potential relevance to clinical populations.

## Introduction

Until recently, cannabis and the bioactive derivative CBD have been used sporadically for decades to treat medically intractable epilepsy.^1,2^ There is now growing scientific and clinical evidence for the antiseizure effects of CBD.^1,3–7^ Currently, pharmaceutical-grade CBD is approved by the U.S. Food and Drug Administration for the treatment of Dravet syndrome and Lennox-Gastaut Syndrome.^1,2^ Additionally, given more widespread legalization and decriminalization worldwide, investigators have begun to explore other potential clinical applications of phytocannabinoids.

While clinicians appreciate that ≥10% of patients can experience short-term treatment-emergent side-effects such as somnolence, diarrhea, convulsions, vomiting, decreased appetite, and fatigue,^2,7–9^ it is uncertain whether CBD presents any long-term safety issues, in particular cognitive and behavioral deficits. The safety concerns of certain phytocannabinoids may be of paramount importance, especially in the developing and adolescent brain, since prior studies have suggested that chronic cannabis exposure can significantly affect brain neurochemistry, anatomy, and physiology, and impair cognition and behavior, especially during brain maturation and in the context of normal cellular processes affecting synaptic plasticity.^9–13^

Children with Dravet syndrome and Lennox–Gastaut syndrome have been reported to respond favorably to CBD at doses of 10–20 mg/kg/day,^1,2,7^ and the current clinical and experimental literature suggests that therapeutic plasma concentrations of CBD may range between 100 nM and 10 μM, with the high nM to very low μM range being most clinically relevant.^1,5,6,8^ In rodents, CBD has antiseizure effects over a broad dose range (5−400 mg/kg)^6^; however, CBD plasma concentrations in earlier studies have not been consistently reported. Thus, the safe therapeutic plasma concentration range of CBD is yet to be clearly defined.

In recent years, linkages between cannabinoids and cellular metabolism have been increasingly established, notably with respect to mitochondrial function.^14–19^ For example, Marsicano and colleagues reported that cannabinoids can impair mitochondrial bioenergetics, and in so doing perturb memory formation.^16^ Hence, we asked whether CBD might exert significant effects on neuronal integrity and brain mitochondrial bioenergetics within a clinically relevant therapeutic concentration range. We found that at low micromolar concentrations, the oxygen consumption rate (OCR) is impaired and there is a lowered threshold for mitochondrial permeability transition (mPT), a critical “death switch” for the cell.^20–22^ We provide evidence that these metabolic changes can potentially contribute to neuronal injury, with subsequent cognitive and behavioral abnormalities, in a dose-dependent manner. Furthermore, these findings correlate with the effects of CBD on a novel chloride conductance on the inner mitochondrial membrane, suggesting a potential mechanism of neurotoxicity.

## Materials and Methods

### Animals

Wistar rats were obtained from the Charles River (Wilmington, MA). Cannabinoid receptor type 1 knockout (CB_1_ KO) mice^23^ were maintained at the University of Calgary. Experiments were performed on male mice and rats postnatal 34–36 (P34–36). This age range was chosen to reflect the stage of rodent brain development comparable with adolescence in humans. All animals were fed *ad libitum* with standard rodent chow (irradiated Pico-Vac Mouse Diet 20; LabDiet, St. Louis, MO) and housed in a humidity-controlled room with a 12-h light/dark cycle. All procedures were approved by the Health Sciences Animal Care Committee of the University of Calgary and carried out in accordance with the Canadian Council on Animal Care Guidelines for the Care and Use of Laboratory Animals.

### Mitochondrial experiments

#### Mitochondrial isolation

Whole-brain mitochondria were acutely isolated from rodents using differential centrifugation, nitrogen disruption, and a Ficoll gradient as previously reported.^24,25^ Following Ficoll purification, the mitochondrial pellet was resuspended in isolation buffer without EGTA and centrifuged at 10,000×*g* for 10 min at 4°C, and then resuspended in 25 μL of isolation buffer without EGTA and stored immediately on ice. For mitoplast preparation, the resulting pellet after ultracentrifugation was resuspended in hypertonic solution (750 mM KCl, 30 mM K^+^-HEPES, 22 mM sucrose, 1 mM EGTA, 0.1 mM CaCl_2_ at pH 7.2) and centrifuged at 10,000×*g* for 10 min. The final pellet was resuspended in 25 μL of hypertonic solution and stored on ice before mitoplast preparation.

#### Mitochondrial bioenergetic assay

A Seahorse Bioscience XFe24 extracellular flux analyzer was used to measure mitochondrial function in cultured cells or intact isolated mitochondria. BE(2)-M17 neuroblastoma cells (density, 35×10^3^) were cultured in microplates under normal conditions for 48 h. Cells were treated with different concentrations of CBD for 24 h. On the experimental day, M17 cells were washed with the XF assay medium supplemented with 25 mM D-glucose, 4 mM l-glutamine, and 2 mM sodium pyruvate and then 500 μL of XF assay medium was gently added to each well. Oligomycin (1 μg/mL), carbonyl cyanide 4-(trifluoromethoxy) phenylhydrazone (FCCP, 0.5 μM), and antimycin A (1 μM) were injected sequentially through ports A–C, respectively.

Acutely isolated brain mitochondria were diluted to the concentration required for plating (5 μg/50 μL) in respiration buffer (125 mM KCl, 2 mM MgCl_2_, 2.5 mM KH_2_PO_4_, 20 mM HEPES, and 0.1% bovine serum albumin [BSA] at pH 7.2) and treated with different concentrations of CBD solution (Sigma-Aldrich, St. Louis, MO) for 10 min. Next, 50 μL of mitochondrial suspension was delivered to each well and the plate was spun at 2,000 rpm for 20 min at 4°C. ADP/pyruvate/malate (4/5/2.5 mM), oligomycin (2 μg/mL), FCCP (4 μM), and antimycin A (2 μM) were injected sequentially through ports A–D, respectively. Relative OCR indicates a percent difference. All data were normalized by either cell number or the amount of mitochondrial protein. Relative OCR (%) reflects “relative respiration to control×100.”

ATP production was calculated from the difference between basal respiration and the value after oligomycin administration. Basal respiration with 15 μM of CBD was found to be very low, and cells exposed to this concentration did not produce any measurable ATP. Thus, the values after oligomycin administration are similar to or slightly lower than the basal amounts in some experiments—hence, resulting in negative values.

#### mPT assay

Mitochondria (100 μg) were placed in 2 mL of respiration buffer in a constantly stirred, temperature-controlled cuvette at 37°C with 100 nM CaG5N (excitation 506 nm and emission 532 nm) and 75 nM TMRE (excitation 550 nm and emission 575 nm) in a Shimadzu RF-5301PC spectrofluorophotometer (Kyoto, Japan). CaG5N was used to monitor extramitochondrial Ca^2+^, and TMRE was used to simultaneously monitor changes in Δψ_m_. Each time-scan began with a baseline reading followed by a 5 mM pyruvate, 2.5 mM malate, and 150 μM ADP addition and at 3 min, Ca^2+^ was added through bolus additions (2 μL of 32 μM CaCl_2_ every 3 min) until the mitochondria were no longer able to buffer the added Ca^2+^.

Data were collected every 2 sec, and sample sizes were chosen based on previously published work utilizing the same experimental methods and approaches.^24^ The spectrofluorophotometer traces were quantified using the Shimadzu Hyper RF software. Mitochondrial Ca^2+^ uptake capacity was calculated as the amount of Ca^2+^ added before the point at which the CaG5N signal was 150% above the mean baseline reading.^26^

#### Mitoplast preparation

Two microliter aliquots of isolated mitochondria were added to 2 mL of hypotonic solution (5 K^+^-HEPES, 1 EGTA, 0.1 CaCl_2_ at pH 7.2) in Falcon 35-mm Petri dishes (Corning, Tewksbury, MA), mixed, and incubated on ice for 1 min to allow the mitochondria to swell, rupturing their outer membranes. The hypertonic solution (500 μL) was then added and mixed to restore isotonicity, resulting in a preparation of swollen inner mitochondrial membranes with the outer membranes removed (mitoplasts) and floating in a solution of 150 mm KCl.

### Electrophysiology

Patch-clamp recordings on mitoplasts prepared from acutely isolated Wistar rat brain and CB_1_ KO mouse brain mitochondria were performed as previously described.^27^ Currents are described using the standard convention, and voltages refer to the inside of the mitoplast (opposite of the command potential). Hippocampal long-term potentiation (LTP) studies were conducted on C3HeB/FeJ mice at P35–40. Transverse hippocampal slices (400 μm) were prepared and a high-frequency LTP protocol was implemented as previously described.^28^

### Morris water maze testing

For spatial learning experiments, male Wistar rats were treated with CBD at three different daily doses intraperitoneally (IP) (30, 100, and 300 mg/kg/day; *N*=9, 10, and 6, respectively) for 12 days beginning at approximately P30. CBD powder was dissolved in vehicle (1:4:15 ratio of Ethanol:Kolliphor EL:0.9% Saline). A separate control group (*N*=6) was given only vehicle. The Morris water maze testing began on day 8 of CBD treatment for all groups at P38. The rats were handled every day for 1 week before testing to familiarize themselves with the experimenter. For the Morris water maze task, a white circular pool measuring 210 cm in diameter was filled with water (22°C±0.5°C) to a level of 2 cm above a hidden platform. Each training trial lasted 60 sec or until the rat located the hidden platform.

If the platform was not located within 60 sec, the rat was manually guided and left there for 10 sec. The time line involved 5 consecutive days of training sessions, with four trials per session. On the 6th day of testing, a probe trial was conducted for one trial lasting 60 sec. After the behavioral test was concluded, blood and brain tissues (hippocampus) were immediately collected and stored at −80°C until liquid chromatography - mass spectrometry (LC-MS)/multiple reaction monitoring (MRM) analysis.

### Analytical measurements of CBD

#### Standard solutions, reagents, and calibration curves

Standard and deuterated internal standard (IS) stock solutions were purchased from Cerilliant (Round Rock, TX). The standard solution of CBD was prepared in 1.0 mg/mL in acetonitrile, and subsequently diluted in 50% methanol/water to produce a set of standards ranging from 0.1 to 100 ng/mL (0.1, 0.25, 0.5, 1, 2.5, 5, 10, 25, 50, and 100). The IS stock solution (CBD-d3) was initially prepared in 0.1 mg/mL in acetonitrile, and subsequently diluted in 50% methanol/water to reach 10 ng/mL. LC-MS-grade acetonitrile, water, and formic acid were purchased from Thermo Fisher Scientific (Edmonton, Canada). Solutions used to establish calibration curves were prepared by mixing 20 μL of each standard solution and 20 μL of IS solution. The calibrators were analyzed in triplicate and the resulting calibration curves were fit by line of regression using a weight of 1/*x*^2^.

#### Sample preparation

Glass tubes (containing 100 μL of IS and 2 mL of acetonitrile) were prepared to receive samples. Plasma samples were thawed at room temperature and pipetted into the prepared tubes; brain (hippocampal) tissue samples were weighed and the frozen piece manually homogenized in the glass tube with a glass rod until it resembled sand. All samples were then sonicated (for 30 min within an ice bath) and stored overnight to precipitate proteins at −20°C. The next day, samples were centrifuged (4°C; 1800 rpm; 3–4 min) to remove particulates, the supernatant was removed from each sample and transferred to a new glass tube, and these tubes were placed under nitrogen gas to evaporate.

Following evaporation, the tube sidewalls were washed with 250 μL of acetonitrile to recollect any adhering lipids and then placed again under nitrogen gas to fully evaporate. Evaporated samples were resuspended in 100 μL of 1:1 methanol and deionized water and underwent two rounds of centrifugation (4°C; 15,000 rpm; 20 min) to remove particulates. The final supernatant was transferred to a glass vial with a glass insert and stored at −80°C until analysis by LC-MS/MRM.

#### Liquid chromatography with tandem mass spectrometry analysis

The LC-MS/MRM analysis was performed at the Southern Alberta Mass Spectrometry (SAMS) facility at the University of Calgary using an Eksigent Micro LC200 coupled with an AB Sciex QTRAP 5500 mass spectrometer (AB Sciex, Ontario, Canada). The data were acquired in positive electrospray ionization and MRM mode. MRM transitions and the collision energy (CE) of all compounds are listed in Figure 7B. CBD-1 and its deuterated standard CBD-d3-1 are the most abundant product ion and used to quantify the compound, referred to as the quantifier; however, in limited cases, the quantifier peak was confounded by nearby peaks, and so, a second product ion (CBD-2 and its deuterated standard CBD-d3-2) was used to confirm the compound, referred to as the qualifier. Ion spray voltage was 5500 V. Nebulizer gas (GS 1), auxiliary gas (GS 2), and curtain gas (CUR) were 30, 30, and 35 AU (arbitrary units).

Declustering potential, entrance potential, and cell exit potential were 80, 7, and 14 V. Quantification of CBD was determined relative to its ratio to the IS and converted to the total content within the 100 μL examined, which was then normalized to the tissue weight or plasma volume of the initial sample.

### Cell viability assay

Murine hippocampal HT22 cells were cultured in Dulbecco's modified Eagle's medium (DMEM) supplemented with 10% fetal bovine serum, 100 U/mL penicillin, and 100 mg/mL streptomycin. HT22 cells were seeded in 96-well culture plates and grown overnight in a humidified incubator with 5% CO_2_ at 37°C. After that, cells were treated with various concentrations of CBD solution for 24 h. Changes in HT22 cell viability after CBD administration were evaluated by the 3-(4,5-dimethylthiazol-2-yl)-2,5-diphenyltetrazolium bromide (MTT) reduction assay. The MTT assay used measuring cell viability was as previously described.^28^

### Chemicals

The CBD solution and lysophosphatidylinositol (LPI) were purchased from Sigma-Aldrich. CBD powder, anandamide (AEA), and AM251 were acquired from Cayman Chemical (Ann Arbor, MI).

### Statistics

For each set of experiments, randomization and blinding were used to the greatest practical extent possible. Graphs and figures were prepared using GraphPad Prism 7.0 (La Jolla, CA) and CorelDRAW X7 (Ottawa, Canada) software. The statistical analysis of the data was performed using GraphPad Prism 7.0 software. The difference between groups was examined by either a one-way analysis of variance (ANOVA) with *post hoc* Tukey test, or a two-way ANOVA with a Šidák *post hoc* test. Values of *p*<0.05 were considered significant.

## Results

### CBD disrupts mitochondrial respiration

**FIG. 1.**
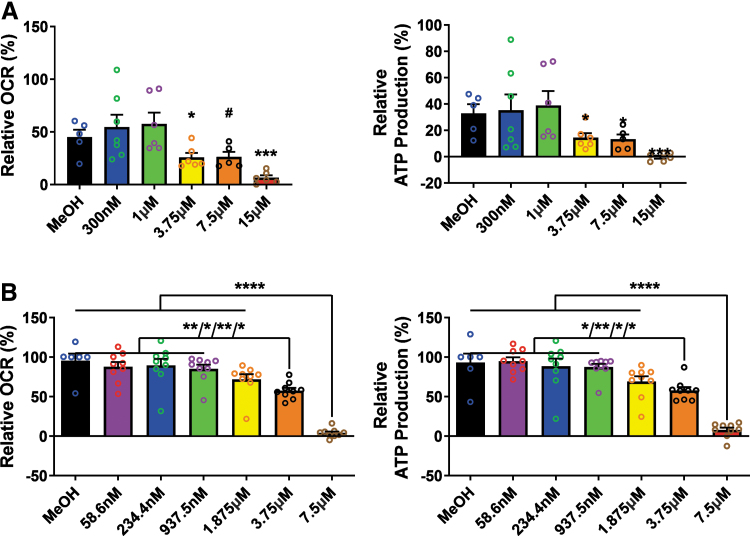
CBD decreases OCRs and ATP production in BE(2)-M17 neuroblastoma cells and in acutely isolated rat brain mitochondria. **(A)** Neuroblastoma cells were treated with CBD (0.3, 1, 3.75, 7.5, or 15 μM) for 24 h. Left panel shows basal respiration and the right panel indicates ATP production. Both basal respiration and ATP production are significantly decreased by CBD at concentrations >1 μM (one-way ANOVA; OCR [F(5, 29)=(6.032), *p*=0.0006] and ATP production [F(5, 29)=(3.718), *p*=0.0101]). Total protein quantity per well was used to normalize the data. Data are expressed as mean±SEM (*n*=2–4 replicate wells in each group from two separate experiments) and *p<0.05, ***p<0.001, and ^#^*p*=0.06. **(B)** Isolated rat brain mitochondria were treated with different concentrations of CBD (0.06, 0.23, 0.94, 1.875, 3.75, or 7.5 μM) for 10 min. Left panel shows basal respiration and right panel indicates ATP production. Both basal respiration and ATP production are significantly decreased by CBD at concentrations >1.875 μM (one-way ANOVA with Tukey *post hoc* test; OCR [F(6, 53)=(28.24), *p*<0.0001] and ATP production [F(6, 53)=(23.72), *p*<0.0001]). Data are expressed as mean±SEM (*n*=3 rats per group) and *p<0.05, **p<0.01, and ****p<0.0001. ANOVA, analysis of variance; CBD, cannabidiol; OCR, oxygen consumption rate; SEM, standard error of the mean.

To demonstrate whether clinically relevant concentrations of CBD can modulate brain mitochondrial function and whether changes induced by CBD on the mitochondria are potentially beneficial or deleterious, we initially evaluated mitochondrial function in BE(2)-M17 neuroblastoma cells that were treated with a range of CBD concentrations. CBD at high nanomolar/low micromolar concentrations resulted in concentration-dependent reductions in the basal OCR and concomitant ATP production in rat hippocampal primary cells (Fig. 1A). CBD, at concentrations ranging from 300 nM to 1 μM, showed a trend toward increased mitochondrial function. However, CBD, at concentrations >3.75 μM, significantly (*p*<0.05) decreased mitochondrial respiration, suggesting mitochondrial dysfunction (Fig. 1A).

Next, to establish the brain mitochondria bioenergetic profile of CBD, we tested mitochondrial respiration in acutely isolated rat brain mitochondria. Consistent with cultured BE(2)-M17 neuroblastoma cells, we found that CBD at concentrations >1.875 μM significantly (*p*<0.05) decreased mitochondrial OCRs, a finding that correlated with decreased production of ATP (Fig. 1B).

### CBD decreases the threshold for mPT activation

**FIG. 2.**
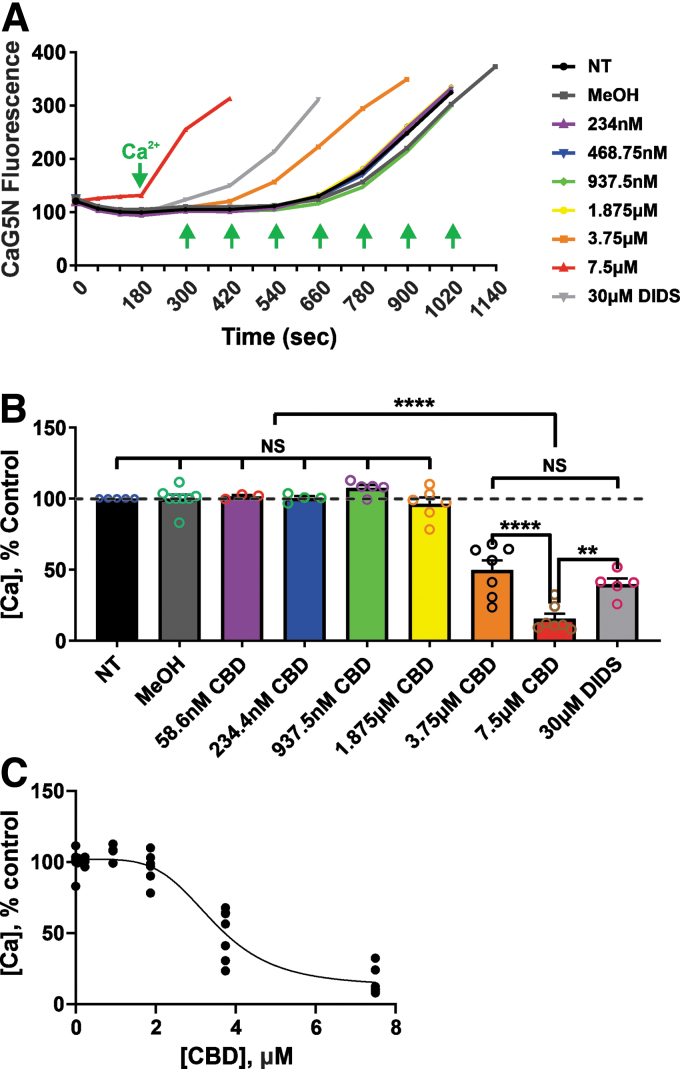
CBD reduces the threshold for mPT and calcium uptake in acutely isolated rat brain mitochondria. **(A)** Representative Ca^2+^ infusion traces demonstrating that CBD, at concentrations >1.875 μM, reduced the threshold for Ca^2+^-induced mPT in acutely isolated rat brain mitochondria. The *y*-axis of the graph is expressed in CaG5N fluorescence AU. **(B)** Summary bar graph reflecting the mean±SEM of mPT thresholds at different CBD concentrations. The *y-*axis indicates the amount of Ca^2+^ added to the mitochondrial suspension. mPT threshold and calcium uptake were significantly reduced by CBD at concentrations >1.875 μM. **(C)** Shows the concentration–response curve for inhibition of calcium uptake by CBD. CBD, at concentrations lower than ∼2 μM, does not affect mitochondrial mPT and calcium uptake. Data are expressed as mean±SEM (*n*=3∼8 rats per group) and NS, **p<0.01, and ****p<0.0001; statistical analysis was performed using one-way ANOVA with Tukey *post hoc* test, [F(8, 41)=(73.42), *p*<0.0001]. AU, arbitrary units; mPT, mitochondrial permeability transition; NS, nonsignificant.

Based on our bioenergetic experiments, we hypothesized that CBD, at concentrations greater than ∼2 μM, might activate the mPT pore. Indeed, we found that CBD altered the threshold for mPT (Fig. 2A). CBD, at concentrations >1.875 μM, reduced the calcium-induced mPT threshold in a concentration-dependent manner, comparable with effects seen with the known chloride channel inhibitor DIDS (4,4′-diisothiochanatostilbene-2,2′-disulfonic acid). Also, CBD reduced calcium uptake in acutely isolated mitochondria from normal rat brain, and DIDS similarly blocked Ca^2+^ uptake. These results provide evidence that CBD at very low micromolar concentrations can disrupt the ability of mitochondria to buffer Ca^2+^ (Fig. 2B, C).

### CBD blocks a novel mitochondrial chloride channel

**FIG. 3.**
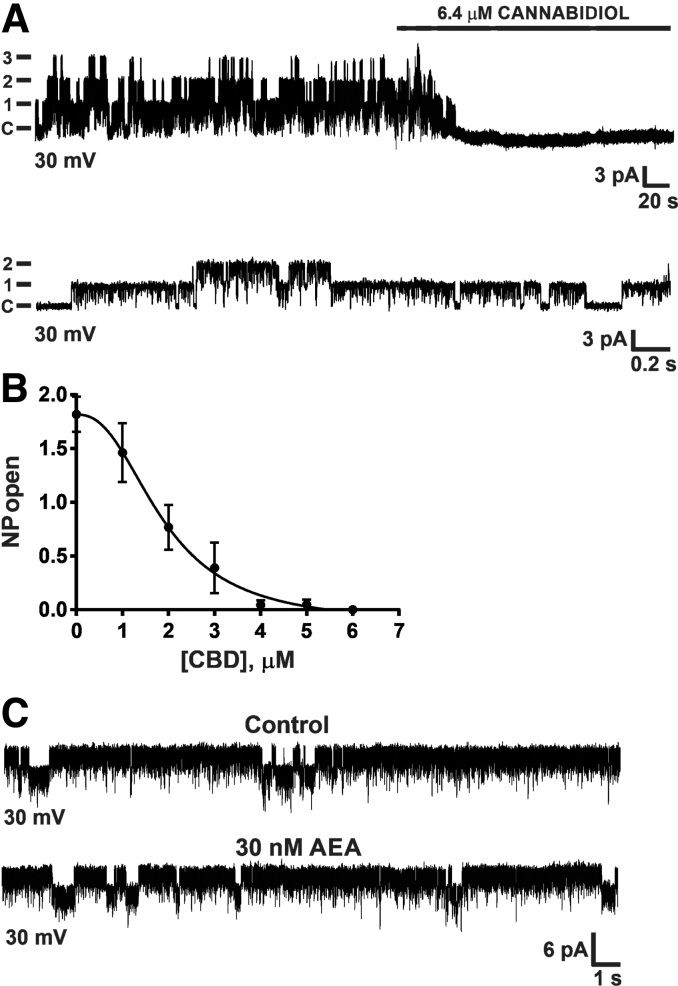
CBD potently blocks a novel mitochondrial inner membrane chloride channel. **(A)** Top graph is a representative mitoplast-attached recording at +30 mV, showing activity of three channels, each with a conductance of ∼100 pS. Addition of 6.4 μM CBD to the bath abolished channel activity. Bottom graph shows that channel activity was partially restored by washout. C=closed state (*n*=3 per group). **(B)** The concentration–response curve for the effect of CBD on the open probability (NP open) of a mitochondrial inner membrane chloride channel from acutely isolated Wistar rat brain mitochondria. At low micromolar concentrations, CBD potently inhibited the chloride channel with an IC_50_ of ∼1.9 μM. Data are pooled from 31 mitoplast recordings and expressed as mean±SEM. **(C)** The endogenous cannabinoid AEA does not affect mitochondrial chloride channel activity. Chloride channel activity before and after AEA. Top graph is baseline channel activity at +30 mV. Bottom graph is channel activity after bath application of AEA (30 nM; +30 mV; *n*=3 per group). AEA, anandamide.

In patch-clamp recordings of mitoplasts prepared from acutely isolated brain mitochondria (from rats aged P35), we observed a chloride-selective conductance of ∼100 pS, as previously characterized.^27^ Bath application of CBD resulted in a dose-dependent inhibition of chloride channel activity with an IC_50_ of ∼2 μM (Fig. 3A, B). Next, we considered whether endogenous cannabinoids such as AEA can modulate mitochondrial chloride (mtCl) channel conductance. We found that AEA (10−50 nM range) did not affect channel activity (Fig. 3C).

### Mitochondrial effects of CBD are independent of CB_1_ receptors

**FIG. 4.**
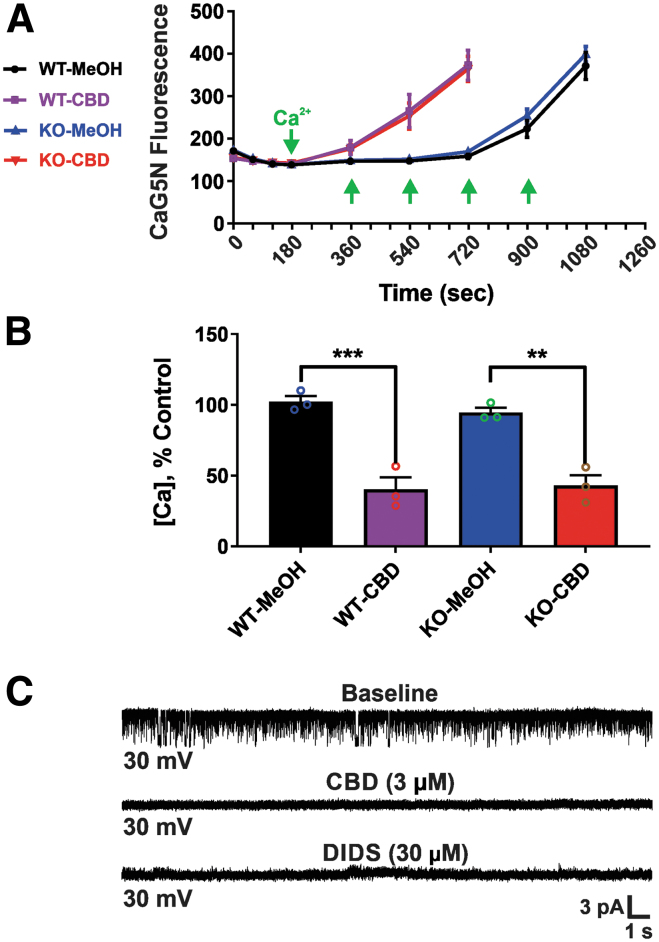
CBD effects are independent of CB_1_ receptors. CBD effects on mPT and mtCl channels are unaltered in acutely isolated mitochondria from CB_1_ KO mice compared with wild-type mice. **(A)** CBD at 3.75 μM significantly decreases the threshold for calcium-induced mPT in both wild-type and CB_1_ KO mice, as observed in normal Wistar rat brain. (Left) Ca^2+^ infusion traces. The *y*-axis of the graph is expressed in CaG5N fluorescence AU. **(B)** Bar graph depicting mean±SEM of mPT threshold (*n*=3 mice per group). The *y-*axis indicates the amount of Ca^2+^ added to the mitochondrial suspension. Data represent the mean±SEM, **p<0.01 and ***p<0.001, one-way ANOVA with Tukey *post hoc* test [F(3, 8)=(28.67), *p*=0.0001]. **(C)** Representative single-channel recording in CB_1_ KO mouse brain mitoplasts (*n*=3 per group) at a holding potential of +30 mV, showing that CBD (3 μM) and DIDS (30 μM) completely block chloride channel activity (indistinguishable from that seen in normal Wistar rat brain). CB_1_ KO, cannabinoid receptor type 1 knockout; DIDS, 4,4′-diisothiochanatostilbene-2,2′-disulfonic acid; mtCl, mitochondrial chloride.

While CBD does not act as a CB_1_ receptor agonist similar to THC, several lines of evidence have indicated that CBD may indirectly increase endocannabinoid signaling at CB_1_ receptors.^29–32^ To assess whether mitochondrial dysfunction induced by CBD could be mediated through mitochondrial CB_1_ receptors, we repeated mPT threshold testing and electrophysiological experiments in acutely isolated mitochondria from CB_1_ KO mice. We found that the effects of CBD on mPT and mtCl channels were unaltered. CBD at 3.75 μM similarly decreased the threshold for calcium-induced mPT threshold in both wild-type and CB_1_ KO mice (reductions of 60.7% and 54.6% with 3.75 μM CBD, respectively), similar to that observed in rat brain (reductions of 50.3% with 3.75 μM CBD) (Fig. 4A, B).

In patch-clamp recordings of CB_1_ KO mouse mitoplasts, spontaneous chloride channel activity was indistinguishable from that seen with rat mitoplasts. In addition, chloride channel activity was completely blocked by 3 μM CBD, and by the chloride channel blocker DIDS (30 μM) (Fig. 4C). These *in vitro* results provide evidence that the observed mitochondrial effects of CBD are not mediated through mitochondrial CB_1_ receptors.

### Mitochondrial effects of CBD are independent of G-protein-coupled receptor 55 receptors

**FIG. 5.**
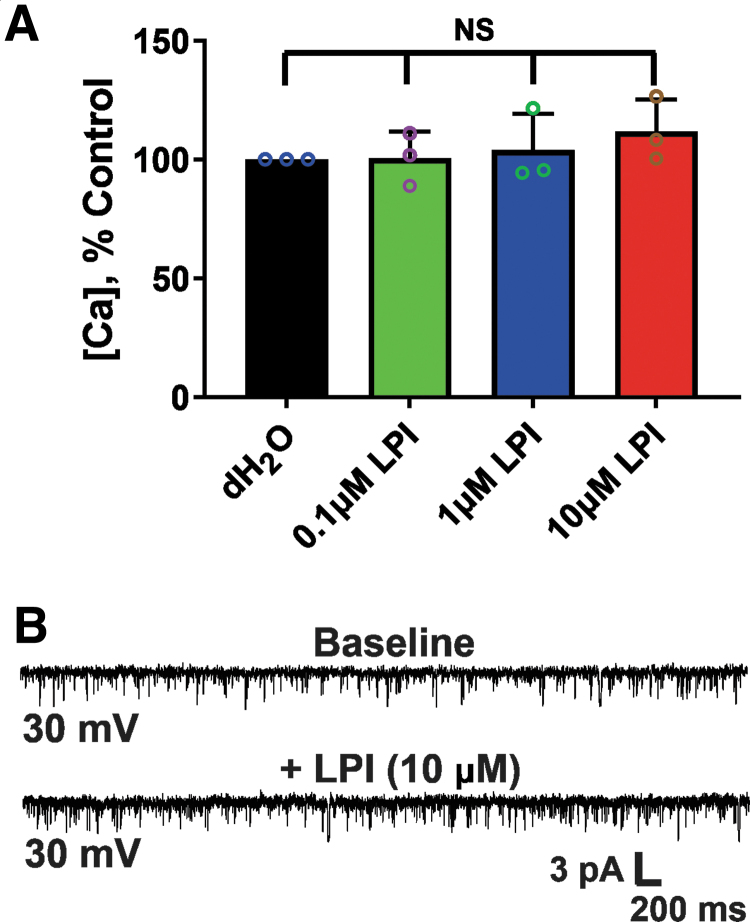
LPI, a GPR55 agonist, does not alter mPT and mtCl channel activity in acutely isolated rat brain mitochondria. **(A)** The threshold for calcium-induced mPT is not affected by LPI at concentrations ranging from 0.1 to 10 μM. Bar graph depicting mean±SEM of mPT threshold (*n*=3 rats per group). The *y*-axis indicates the amount of Ca^2+^ added to the mitochondrial suspension. One-way ANOVA [F(3, 8)=(0.6527), *p*=0.6033, NS]. **(B)** Representative recordings at +30 mV showing mitochondrial chloride channel activity before (top graph) and after 10 μM LPI (bottom graph). Channel activity was unaffected by 10 μM LPI (*n*=3 per group). Downward deflections indicate channel openings. GPR55, G-protein-coupled receptor 55; LPI, lysophosphatidylinositol.

The G-protein-coupled receptor 55 (GPR55) has been shown to cause Ca^2+^ release from intracellular stores through activation by its endogenous agonist LPI,^33^ raising the possibility that a mitochondrial GPR55 could be involved in the observed effects of CBD. To address this possibility, we tested the effects of LPI (0.1, 1, or 10 μM) on the mPT threshold and mtCl channels in acutely isolated brain mitochondria from rat brain. This range of LPI concentrations was chosen based on the known EC_50_ values in various *in vitro* systems.^34,35^ We found no effects of LPI on either the mPT threshold or chloride channel activity (Fig. 5), consistent with the view that GPR55 is not involved in the observed effects of CBD at the mitochondrial level.

### CBD causes synaptic potentiation, learning, and memory impairment

**FIG. 6.**
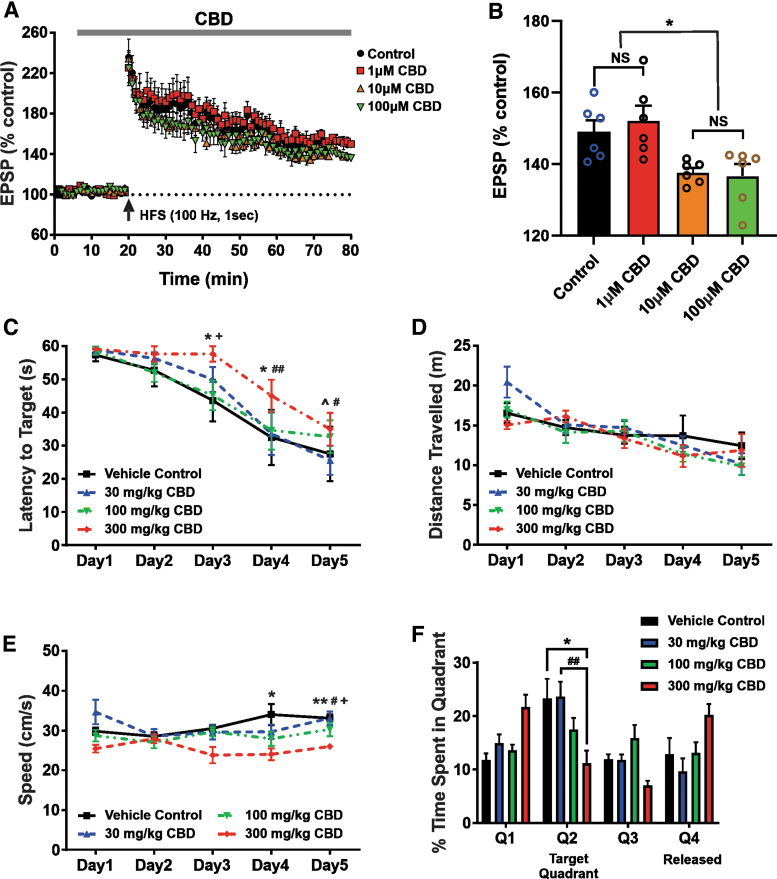
High CBD doses exert impairment of hippocampal LTP and decrease spatial learning and memory in rodents. **(A, B)** CBD impairs hippocampal LTP in a concentration-dependent manner. **(A)** LTP recorded in hippocampal slices of C3HeB/FeJ mice administered to various concentrations of CBD. HFS (100 Hz, 1 sec) of Schaffer collaterals in CA1 hippocampus led to intact LTP formation when either physiological saline or 1 μM CBD was perfused. However, 10 and 100 μM CBD resulted in inhibition of LTP maintenance. The vertical arrow in this figure indicates the time point of HFS initiation. The dotted line denotes the average of the baseline EPSP amplitude during a 10-min physiological saline infusion before HFS stimulation. **(B)** Summary of changes in EPSP amplitudes at 60 min after HFS among the experimental groups (*n*=6 per group). Each vertical bar represents the mean EPSP amplitude±SEM. NS, *p<0.05. A one-way ANOVA revealed a significant effect of CBD concentration on hippocampal LTP [F(3, 20)=(6.2558), *p*=0.0036]. A Tukey *post hoc* test revealed that CBD at concentrations of 10 and 100 μM significantly impairs hippocampal LTP compared with 1 μM CBD (*p*=0.0193 and *p*=0.0103, respectively), but not controls (*p*=0.0937 and *p*=0.0534, respectively). **(C–F)** High dose of CBD treatment decreases spatial learning and memory and results in slower swimming speeds of Wistar rats. Rats were injected daily with three different concentrations of CBD (30, 100, and 300 mg/kg) for 12 days. **(C)** A one-way ANOVA revealed a significant effect of treatment on latency to target quadrant [F(3, 12)=(6.534), *p*=0.0072]. A Tukey *post hoc* test revealed that the spatial learning and memory of animals treated with 300 mg/kg CBD were significantly impaired on days 3, 4, and 5 compared with the other groups. **(D)** The average distance traveled per training day, with no significant differences. **(E)** The average swimming speed per training day, demonstrating that the 300 mg/kg treatment group swam significantly slower on training days 4 and 5. A one-way ANOVA revealed a significant effect of treatment on the average swimming speed [F(3, 12)=(8.995), *p*<0.0021]. A Tukey *post hoc* test revealed that the 300 mg/kg treatment group swam significantly slower on training days 4 and 5. **(F)** A one-way ANOVA revealed a significant effect of treatment on time spent in the target quadrant [F(3, 27)=(4.011), *p*=0.0175]. A Tukey multiple comparisons test revealed that the group treated with 300 mg/kg CBD spent significantly less time in the target quadrant (*M*=11.2, SD=5.8 sec) than the vehicle control (*M*=23.3, SD=8.9 sec) and 30 mg/kg groups (*M*=23.6, SD=5.6 sec), but not the 100 mg/kg group (*M*=17.5, SD=5.4 sec). Data represent the mean±SEM. ^*p*>0.05 between vehicle control and 30 mg/kg; *p<0.05 between vehicle control and 300 mg/kg; **p<0.01 between vehicle control and 300 mg/kg; ^#^*p*<0.05 between 30 mg/kg and 300 mg/kg; ^##^*p*<0.01 between 30 mg/kg and 300 mg/kg; ^+^*p*<0.05 between 100 mg/kg and 300 mg/kg. EPSP, excitatory postsynaptic potential; HFS, high-frequency stimulation; LTP, long-term potentiation; SD, standard deviation.

Importantly, to determine the functional consequences of CBD-induced changes in mitochondrial function, we conducted further cellular electrophysiological and behavioral experiments. LTP in the hippocampal CA1 region has been extensively used to study the mechanisms of learning and memory.^36^ When hippocampal slices were treated with 1 μM CBD, no differences were seen in the excitatory postsynaptic potential (EPSP) amplitude between the control and CBD-applied groups at 60 min post-high-frequency stimulation (HFS). In contrast, in hippocampal slices exposed to 10 and 100 μM CBD, the EPSP amplitude at 60 min post-HFS was significantly reduced compared with either the control or 1 μM CBD group (Fig. 6A, B).

Next, the Morris water maze task was used to assess the spatial learning and memory function of animals. When injected daily with a high dose of CBD (300 mg/kg), the rats had significantly (*p*<0.05) higher latencies on training days 3, 4, and 5 (Fig. 6C), when compared with the other groups. Even though the mean distance traveled was not significantly different among the groups (Fig. 6D), the mean speed traveled by the 300 mg/kg CBD treatment group was significantly slower on training days 4 and 5 (Fig. 6E). In addition, during the probe trial, the 300 mg/kg treatment group spent significantly (*p*<0.05) less time in the quadrant where the hidden platform would have been (Fig. 6F).

### Blood and brain concentrations of CBD

**FIG. 7.**
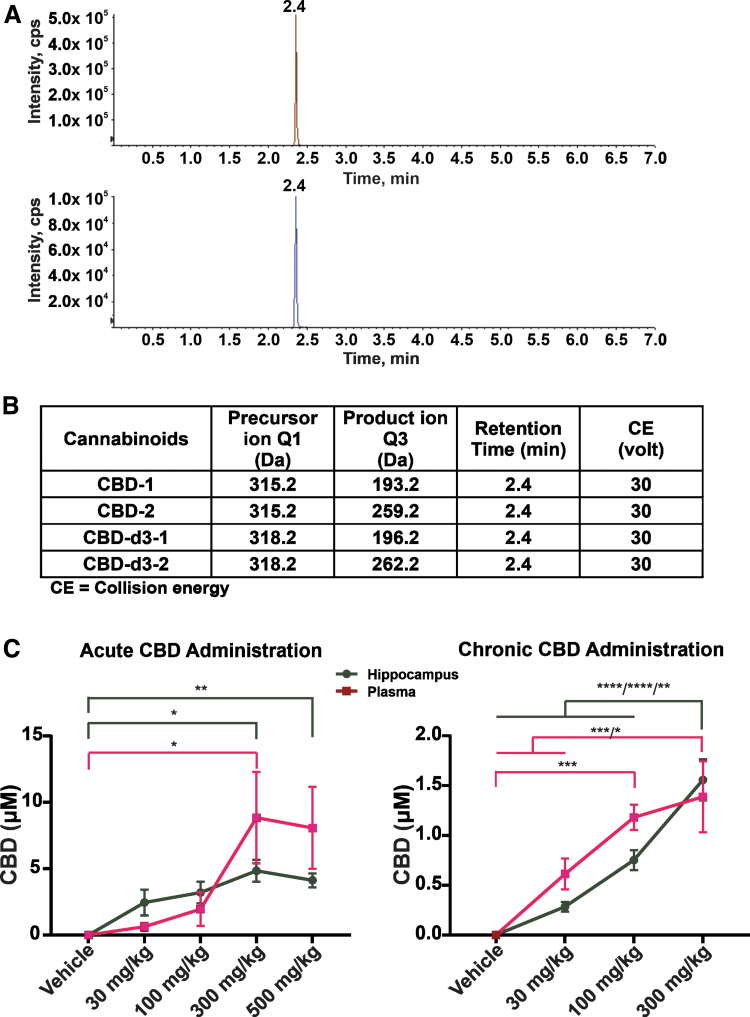
Concentration of CBD in plasma and hippocampus. CBD was administered acutely (30, 100, 300, and 500 mg/kg) or daily (30, 100, and 300 mg/kg) for 12 days by intraperitoneal injection. **(A)** Extracted ion chromatogram of CBD (red) and CBD-d3 (blue). **(B)** MRM transition and CE of compounds. **(C)** CBD concentration in blood and brain (hippocampus) after acute or chronic injection of CBD. For acute CBD administration, *n*=4 male Wistar rats per group. For chronic CBD administration, vehicle control (*n*=6); 30 mg/kg (*n*=9); 100 mg/kg (*n*=10); 300 mg/kg (*n*=6). Data are expressed as mean±SEM. *p<0.05; **p<0.01; ***p<0.001; and ****p<0.0001. Statistical analysis was performed using a two-way ANOVA with a Šidák *post hoc* test [F(3, 47)=(41.20), *p*<0.0001]. CE, collision energy; MRM, multiple reaction monitoring.

In the group of mice receiving 30 mg/kg CBD acutely, the plasma and hippocampal levels were 0.63 and 2.45 μM, respectively. However, chronic CBD administration resulted in peak plasma and hippocampal levels of 0.61 and 0.28 μM, respectively. Acute and chronic intraperitoneal (i.p.) injection of CBD at 100 mg/kg and 100 mg/kg/day, respectively, resulted in plasma levels of 1.96 and 1.18 μM, respectively (Fig. 7C). These values overlap with the lower end of the *in vitro* concentration range that was used for the bioenergetic and electrophysiology experiments detailed above. With respect to the animals administered 300 mg/kg CBD, it was not possible to obtain accurate concentration values because these were above the limit of quantification for the LS/MS-MS system utilized.

### CBD induces neurotoxic effects

**FIG. 8.**
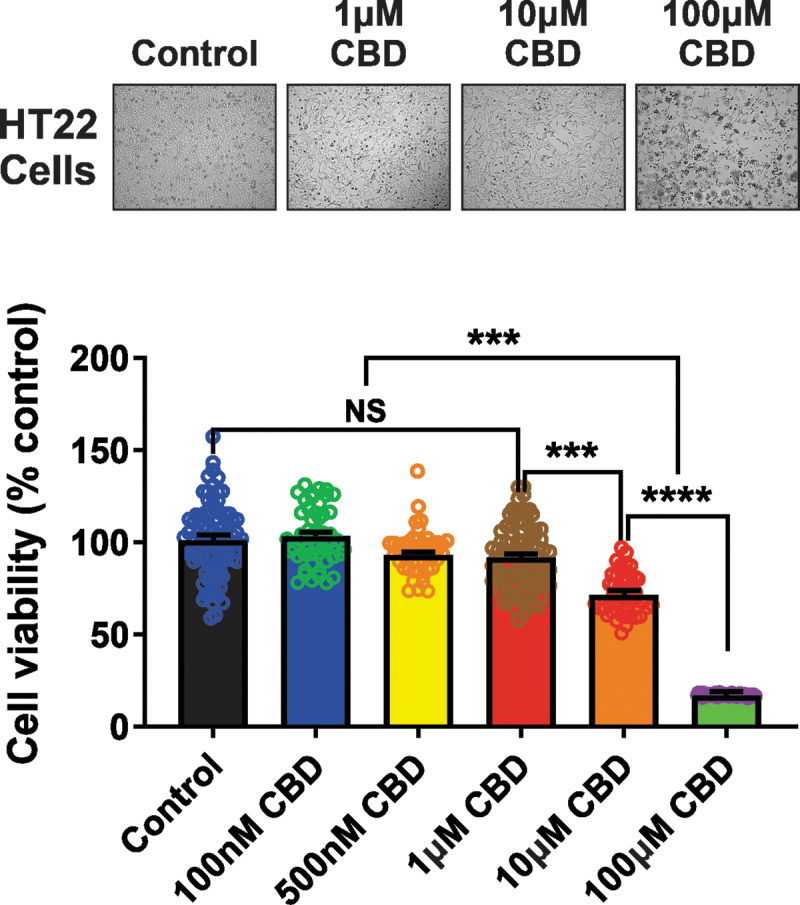
CBD affects cellular viability. Representative photomicrographs (top panel) of HT22 cells after 24 h with media alone or the CBD dose range of 1–100 μM. Summary bar graph (bottom panel) indicating changes in cellular viability at 24 h after the treatment of diverse doses of CBD was expressed as a percentage of the control group. The administration of concentrations ranging from 100 nM to 1 μM CBD did not alter cell viability compared with control. CBD prominently produced neuronal cell death at concentrations >1 μM. A one-way ANOVA revealed a significant effect of CBD concentration on the viability of HT22 cells [F(5, 369)=(79.21), *p*<0.0001]. A Tukey *post hoc* multiple comparisons test revealed that concentrations of CBD >1 μM significantly decrease the viability of HT22 cells (*p*<0.0001) compared with the other groups. Data represent the mean±SEM. NS, ***p<0.001, and ****p<0.0001.

To further explore evidence of CBD neurotoxicity, we assessed changes in the viability of murine hippocampal HT22 cells exposed to CBD in the MTT reduction assay. Application of CBD (1–100 μM) resulted in a significant reduction in cellular viability compared with cells treated with media alone. Notably, HT22 cells exhibited a higher decrease in cellular viability to 16.9%±0.09% of control after a 24-h exposure following treatment with 100 μM CBD. However, concentrations of 100 or 500 nM CBD did not alter the viability of HT22 cells (Fig. 8).

## Discussion

In the present study, we demonstrate that low micromolar concentrations of CBD (≥1–3.75 μM) can impair brain mitochondrial function—possibly through its inhibitory effects on a recently described chloride conductance on the inner mitochondrial membrane in rodent brain (IC_50_ 1.9 μM).^27^ Specifically, within and above this concentration range, CBD profoundly inhibits ATP production, reduces the threshold for Ca^2+^-induced mPT activation, and inhibits mitochondrial Ca^2+^ uptake (IC_50_ 3.5 μM), effects that can collectively induce cellular injury and apoptotic neurodegeneration. Furthermore, we found that CBD can disrupt hippocampal LTP and the Morris water maze performance at low micromolar concentrations and at the high dose (300 mg/kg/day) used, respectively.

Taken together, our data indicate that there exists a critical concentration- and dose-dependent threshold, above which CBD can induce both anatomical and functional neuronal injuries. Our findings build on previous studies,^15,16,19^ and are of clinical relevance as adverse central nervous system effects have not infrequently been reported in CBD clinical trials.^1,2^

Given these results, it is natural to ask whether the other prominent cannabinoid, THC, might induce similar effects. Indeed, THC has also been shown to disrupt mitochondrial respiration in a pulmonary transformed cell line at concentrations as low as 8 μM^37^ and in pig brain mitochondria in the low micromolar range with an IC_50_ of ∼15 μM,^17^ possibly through mitochondrial CB_1_ receptor activation^38^ or through indirect modulation of the mPT pore,^37^ similar to that observed in the current study. However, while there may be an overlap in the threshold concentrations for mitochondrial impairment between CBD and THC, the mechanism(s) responsible for these effects remain unclear. In this context, it is important to note that CBD does not interact with either the CB_1_ or CB_2_ receptors.

### Neurotoxicity of CBD

A few studies have examined the toxicity attributes of CBD. In humans, Epidiolex^®^, an FDA-approved CBD formulation for the treatment of Dravet syndrome and Lennox–Gastaut syndrome, can cause numerous side effects over the recommended dose range of 5–20 mg/kg/day.^39^ The most commonly reported adverse effects include somnolence, decreased appetite, diarrhea, elevations in liver transaminase enzymes, fatigue/malaise/asthenia, rash, insomnia, sleep problems, and a propensity for infections.

Furthermore, while the developmental risks associated with Epidiolex use in pregnant women are unknown, results from pre-clinical studies indicate that chronic oral administration of 250 mg/kg/day of CBD to pregnant rats increases the embryo–fetal mortality rate, whereas 150 or 250 mg/kg/day of long-term exposure throughout pregnancy impacts lactation, decreases growth, delays sexual maturation, produces neurobehavioral changes, and perturbs male reproductive organ.^9^ In this regard, we observed neurotoxic effects over a similar *in vivo* dosage range.

We examined CBD's effects on standard hippocampal-dependent learning and memory paradigms and observed that CBD impaired LTP in a dose-dependent manner at concentrations of 10 μM and higher. Consistent with these observations, the Morris water maze testing revealed that animals exposed to the higher dose range of CBD (300 mg/kg/day) had increased latencies to target, reduced swimming speeds, and reduced time in the quadrant from which the hidden platform had been removed. Taken together, our results indicate that CBD, at high doses, can impair learning and memory function.

In agreement with a recent study,^40^ we found that at lower doses (≤ 100 mg/kg/day), CBD had no influence on spatial learning and long-term memory function in healthy mice. As a final measure of potential cytotoxicity, we exposed hippocampal HT22 cells to various concentrations of CBD for 24 h and found that concentrations ≥10 μM produced prominent neuronal cell death, in concordance with our results showing that CBD induces mitochondrial dysfunction and block of mtCl channel activity over the same concentration range.

### Clinically relevant concentration range of CBD

While delineating *in vivo* dosing in animals or humans is of paramount importance to ensuring both the efficacy and tolerability of any pharmacological agent, the standard parameter for titrating dosing is the serum concentration within an accepted clinically relevant range. In this light, the human pharmacokinetics of CBD have been examined in numerous studies,^41–50^ which have used widely variable doses and routes of administration. Five-to-800 mg of CBD, delivered through smoking, nebulizer, injection, oral, and sublingual routes, has yielded mean blood or plasma concentrations ranging from ∼1 nM to 2 μM at 3 min to 4.5 h postdose.

In an open-label study of pharmaceutical-grade synthetic CBD oral solution in pediatric patients with treatment-resistant epilepsy, a multiple-dose regimen of 10–40 mg/kg/day produced mean peak plasma concentrations of 91–314.5 ng/mL (0.29–1.01 μM).^51^ Moreover, in the 40 mg/kg/day CBD dosing group, concomitant clobazam use increased the mean plasma concentration more than twofold, from 192.9 to 453.7 ng/mL (0.61–1.43 μM), which may be explained by the fact that both CBD and clobazam are metabolized by CYP3A4.^49^ Another study examined the pharmacokinetics of CBD in children with refractory epileptic encephalopathy.^50^ Patients receiving 5.3–19.4 mg/kg/day had a mean (range) peak plasma concentration of 49.6 (14.4–302) ng/mL or 0.16 (0.04–0.96) μM.

Patients who received concomitant levothyroxine (a potent inhibitor of the cytochrome P450 enzyme CYP3A4) showed a fourfold increase in CBD concentration, and were the only patients showing CBD concentrations >200 ng/mL (0.65 μM).^49^ Together, these results imply that more aggressive clinical dosing of CBD, as well as concomitant treatments that are metabolized by, or inhibit, P450 enzymes, can result in plasma concentrations that could potentially impair brain mitochondria and induce neuronal injury.

Although the human brain concentrations following CBD administration are unknown, oral or intraperitoneal doses of 120 mg/kg in rats resulted in ∼2.2–4.3 times higher concentrations in the brain than plasma, depending on the solvent used and the route of administration.^52^ Thus, this approximate two- to fourfold difference between plasma and brain may result in an effective concentration above 3 μM, a level where we have documented mitochondrial and neuronal impairment in the present study. The observation that brain concentrations are significantly higher than those seen in plasma is likely due to rapid passage through the blood−brain barrier and accumulation of CBD in the highly lipid environment of the brain. Thus, when considering the blood levels of CBD, it is important to recognize that central levels may be much higher.

Finally, the actual doses and blood−brain concentrations of CBD in patients using various artisanal formulations of this compound are unknown, and hence, some individuals may potentially be exposed to even greater neurotoxic levels when not consuming pharmaceutical-grade CBD. Collectively, pharmacokinetic studies support the possibility of human brain concentrations reaching those required to elicit the *in vitro* mitochondrial impairment described in the current study.

### Mechanism of neurotoxicity

Stemming from our observation that CBD exerts neurotoxic effects within and above a clinically relevant concentration range, we sought to determine an underlying mechanism, one that could potentially be targeted to mitigate adverse side effects. Such a combined therapeutic approach is not unusual. For example, isoniazid treatment of tuberculosis is usually combined with N-acetyl-cysteine, the latter to prevent adverse effects of the former.

CBD has been shown to reduce seizure activity in various rodent seizure and epilepsy models,^53,54^ but the exact mechanism(s) responsible for this effect remain unclear. To exclude the involvement of CB_1_ receptors in the observed *in vitro* effects of CBD, we repeated the mPT threshold and mitoplast patch-clamp experiments in acutely isolated brain mitochondria from CB_1_ receptor KO mice, and found no differences compared with controls. Overall, in our hands, CBD's effects on the brain mitochondria, and specifically the blockade of mtCl channels, are not dependent on mitochondrial CB_1_ receptors.

Earlier, it was shown that in a mouse model of Dravet syndrome, CBD's antiseizure effects may be mediated through the GPR55 signaling pathway.^53^ GPR55 is primarily expressed in the striatum, cortex, and hippocampus, as well as in the peripheral nervous system,^55^ areas that have been linked to the major phenotypic features of Dravet syndrome, and GPR55 activation enhances intracellular calcium release in neurons.^55^ To investigate the involvement of GPR55 in the observed effects of CBD at the mitochondrial level, we applied the GPR55 agonist LPI to rat mitochondria during mPT threshold testing and mitoplast patch-clamp recordings. LPI-mediated activation of GPR55 had no effect on the mPT threshold or chloride channel activity, suggesting that GPR55 is unlikely to be involved in the observed effects of CBD at the mitochondrial level.

Intriguingly, however, we found that CBD inhibited a recently described mtCl conductance^27^ at low micromolar concentrations, while the endogenous cannabinoid AEA (30 nM) had no effect on this channel. Mitochondrial ion channels have been previously implicated in the mechanism of action of CBD.^56^ In our study, we observed several effects on the mitochondrial function, all tied to the inner mitochondrial membrane, in addition to the cellular and behavioral effects, which would be predicted from the observed mitochondrial impairment. We found that low micromolar CBD blocked a novel chloride channel, reduced the Ca^2+^ activation threshold for mPT, and reduced both the OCR and ATP production.

Interestingly, both CBD and the chloride channel blocker DIDS were able to: (1) block the inner membrane chloride channel; and (2) reduce the threshold for mPT, suggesting a close link between chloride channel inhibition and mPT activation. Because activation of mPT would be predicted to lead to the observed impairment of OCR and ATP production, in addition to the described cellular toxicity, it is possible that CBD acts by inducing mPT activation through chloride channel blockade. A drawback of the current studies is the fact that the molecular identity of the mtCl conductance is unknown, and that there are no selective activators of this channel.

Hence, more definitive mechanistic studies strengthening the causal relationships between the mitochondrial effects observed and the morphological and functional neurotoxicity of CBD could not be pursued. In addition, it is unclear whether any of the known CBD metabolites^52^ may be responsible for the concentration-dependent mitochondrial toxicity, but these investigations are beyond the scope of the present study.

Although there appear to be multiple types of mtCl channels, and while the precise molecular compositions and identities of these channels remain unclear, it is likely that they play an important role in both normal physiology and pathological states by regulating the mitochondrial membrane potential (Ψ), mPT pore, apoptosis, and even mitochondrial fusion and fission processes.^20,21^ A chloride-selective channel on the inner mitochondrial membrane, which is active at physiological (negative) Ψ, could play a critical role in mitochondrial function. Such a conductance would tend to depolarize the Ψ (in opposition to the proton pump) and could serve as a homeostatic regulator of Ψ, membrane structural integrity, and ultimately, ATP production. Blockade of this channel by CBD could lead to a pathological overhyperpolarization of Ψ, which could disrupt membrane structural integrity, potentially leading to mPT activation and the subsequent loss of Ψ.

These effects would in turn lead to impaired respiration and neuronal injury, in line with our observations of the effects of CBD on mitochondrial function, cell viability, and learning and memory function. While the channel described here is active at positive Ψ, this voltage dependence is based on the widespread assumption that mitoplasts form from inner mitochondrial membranes in a matrix-toward-the-inside configuration. However, the precise composition and configuration of these vesicles are unknown, and any reversal of membrane orientation during mitochondrial swelling would lead to an apparent reversal of voltage dependence for the channel. Furthermore, the behavior of the channel under normal physiological conditions *in vivo*, or in pathologic states such as epilepsy, remains unclear.

### Summary

In conclusion, the present study describes the neurotoxicity of CBD above a defined concentration threshold and identifies a potential novel mechanism for this deleterious effect on the neuronal structure and function. Our results have significant implications for clinical utilization and public consumption of CBD and CBD-containing products. Further investigations are necessary to firmly establish both the therapeutic and toxic dose and concentration ranges for CBD in clinical populations, and to elucidate the underlying mechanisms.

## Abbreviations Used

AEA: anandamide
ANOVA: analysis of variance
AU: arbitrary units
CB_1_ KO: cannabinoid receptor type 1 knockout
CBD: cannabidiol
CE: collision energy
DIDS: 4,4′-diisothiochanatostilbene-2,2′-disulfonic acid
EPSP: excitatory postsynaptic potential
FCCP: carbonyl cyanide 4-(trifluoromethoxy) phenylhydrazone
GPR55: G-protein-coupled receptor 55
HFS: high-frequency stimulation
IS: internal standard
LPI: lysophosphatidylinositol
LTP: long-term potentiation
mPT: mitochondrial permeability transition
MRM: multiple reaction monitoring
mtCl: mitochondrial chloride
MTT: 3-(4,5-dimethylthiazol-2-yl)-2,5-diphenyltetrazolium bromide
NS: nonsignificant
OCR: oxygen consumption rate
P: postnatal
SD: standard deviation
SEM: standard error of the mean
THC: Δ^9^-tetrahydrocannabinol
